# Shedding light on optical cochlear implant progress

**DOI:** 10.15252/emmm.202012620

**Published:** 2020-07-26

**Authors:** Siân R Kitcher, Catherine JC Weisz

**Affiliations:** ^1^ Section on Neuronal Circuitry National Institute on Deafness and Other Communication Disorders NIH Bethesda MD USA

**Keywords:** Synthetic Biology & Biotechnology, Neuroscience

## Abstract

Electrical cochlear implants (CI) currently lack the frequency and intensity resolution to allow detection of complex sounds in background noise. The use of microscale optoelectronics in conjunction with optogenetics provides a promising direction in CI technology to allow improvements in spectral resolution, providing a richer soundscape for users. The present work offers the first instance of using multi‐channel μLED‐based optical CI to demonstrate optogenetic activation of auditory neurons.

## Graphical abstract

Kitcher and Weisz highlight implantable optogenetic cochlear implants (oCI) that demonstrate small scale μLED‐based light sources that successfully evoke activity in auditory neurons with the ability to encode sound intensity, and improved spectral selectivity.

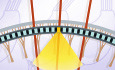

An estimated 466 million individuals worldwide have disabling hearing loss (World Health Organization). Many causes of hearing loss lead to death of sensory hair cells but an otherwise intact neural pathway, allowing use of electrical cochlear implants (eCI) to directly stimulate the auditory nerve. Adults, children, and infants can be safely implanted with eCI, and for individuals with profound hearing loss, they represent a revolution in hearing assistance technology, allowing perception of speech and other sounds.

The complex structure and function of the cochlea make auditory stimulation with electrodes challenging. The cochlea is organized tonotopically, so that hair cells and neurons at the base respond to high‐frequency sounds and those at the apical end respond to low frequencies. A narrower cochlear activation area (Fig 11A) corresponds to more precise pitch perception (spectral selectivity). In eCI users, an array of electrodes is inserted into the cochlea, and the spiral ganglion neurons (SGN), which comprise the auditory nerve, are electrically activated. However, currents from each electrode spread out to activate many neurons (Fig 11B), limiting the spectral selectivity. These physical limitations of eCI result in poor speech perception in background noise and difficulty appreciating complex sounds such as music.

**Figure 1:**
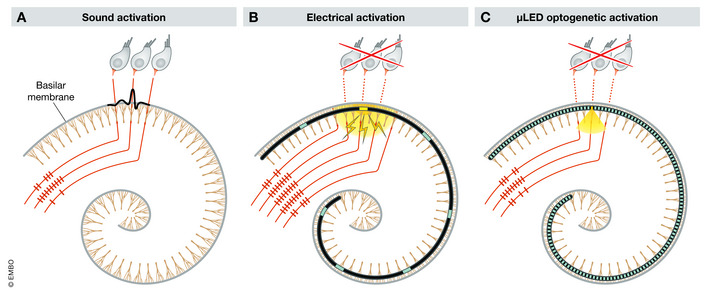
Sound vs cochlear implant‐based auditory nerve activation (A–C) Schematic of mammalian cochlea showing basilar membrane (gray coil), individual spiral ganglion neurons (SGN, light brown lines), and three representative inner hair cells. Red lines represent axons of single SGN projecting to the brain, action potential (AP) patterns evoked by stimulation depicted by short lines on axons. (A) Sound induces the basilar membrane traveling wave (black), deflecting hair cell stereocilia and evoking AP in a limited number of SGN. (B) Cochlea with implanted electrical cochlear implant (eCI), with individual electrodes (blue) and electrical current spread around an electrode (yellow) activating a wider range of SGN. (C) Cochlea with implanted optical cochlear implant (oCI), individual μLEDs (blue), and illumination spreading from a μLED (yellow) activating a limited number of SGN.

Optical cochlear implants (oCI) may overcome the inherent limitations of current spread from eCI electrodes as light can be focused to activate a narrow band of SGN (Fig 11C) (Dombrowski *et al*, 2019). Optogenetics (Boyden *et al*, 2005) has emerged as an effective method for introducing light sensitivity in neurons, and studies from multiple laboratories have demonstrated virus‐mediated expression of fast channelrhodopsin (ChR) variants such as CatCh in SGN. Activation of SGN via optical fibers (Hernandez *et al*, 2014; Duarte *et al*, 2018; Keppeler *et al*, 2018) has been measured up to hundreds of Hz *in vitro* (Keppeler *et al*, 2018), indicating that optogenetic tools have the speed and power for appropriate auditory system stimulation.

To fulfill clinical potential, oCIs will require an array of tiny light sources for focal illumination of the light‐responsive cochlea to evoke spectrally precise SGN activity. In the current manuscript, Dieter *et al* (2020) make a major step toward clinical oCI use by combining optogenetics in SGN with an implanted linear array of microscale thin‐film light‐emitting diodes (μLED). First, SGN were made light‐sensitive with viral expression of CatCh in the cochlea of adult gerbils, and efficacy of optogenetic activation was determined using a laser‐driven optical fiber and auditory brainstem response (ABR) measurement.

To measure the precision of μLED oCI activation of SGNs, the authors employed *in vivo* multielectrode recordings from neurons in the central nucleus of the inferior colliculus (ICC), an integral brain region for sound processing easily accessed at the dorsal surface of the brain (Hernandez *et al*, 2014; Dieter *et al*, 2019). ICC neurons are arranged tonotopically, so activity at different positions along a linear electrode array indicates frequency‐specific auditory system activity. At least four weeks after CatCh injection, the authors implanted a linear 32‐electrode array into the ICC of hearing and kanamycin‐deafened gerbils. The position was confirmed in the hearing gerbils using sound, and equivalent tonotopic slopes applied to recordings from deafened gerbils.

Following ICC multielectrode insertion, oCIs were implanted. oCIs used were a linear array of 16 individually controlled μLEDs separated by 100, 150, or 250 μm center‐to‐center distance. Each μLED measured 60 × 60 μm embedded in biocompatible epoxy and medical grade silicone. The μLEDs were composed of gallium nitride (GaN), emitted at wavelengths optimal for ChR activation, and were optimized for the low power requirements and minimal heat generation required for long‐term implantation (Klein *et al*, 2018). oCIs were implanted in the cochlea either via the round window or by cochleostomy in the basal or middle turn (high‐ and low‐frequency regions, respectively). The orientation was confirmed postmortem with X‐ray tomography.

The oCI μLEDs were illuminated individually or in groups of 4 or 16, while the responses of ICC neurons were recorded and quantified by the cumulative discrimination index (*d*’) (Dieter *et al*, 2019). Increased illumination from single or multiple μLEDs corresponded with increased ICC neuron activity. Single μLEDs at maximum intensity evoked neural responses in ~ 1/3 of ICC neurons, a number increased by stimulating four or 16 μLEDs. Spectral selectivity of oCIs was assessed by measuring the spread of activity across the ICC tonotopic axis in response to illumination of blocks of four adjacent diodes and was found to be comparable to the spectral selectivity of focal optical fiber stimulation. Both have improved spectral selectivity compared to clinical style rodent eCIs (Dieter *et al*, 2019). Increasing radiant flux from individual μLEDs, such as with micro‐lenses (Klein *et al*, 2019), paired with enhanced optogenetic performance through more powerful ChR variants or enhanced viral delivery is expected to improve the responses to illumination from individual μLEDs, thus further improving spectral selectivity.

This work is the first demonstration of implantable oCIs displaying the physical and optical requirements for optogenetic activation of the auditory nerve. Future steps include evaluating the safety of long‐term implantation, improving implant flexibility, and testing animals’ behavioral responses to oCIs activated by sound to assess auditory perception. It is unlikely oCIs will achieve the intensity coding of a hearing ear due to the lack of efferent gain control, but improvements in reliability of SGN activation by individual μLEDs and enhanced single μLED intensity will improve frequency and intensity resolution by allowing use of many more μLEDs to mimic sound‐driven SGN fiber recruitment. Differential expression of opsins to mimic high‐ and low‐spontaneous rate SGNs in the hearing cochlea could also contribute to increased dynamic range once improved viral transduction rates are achieved. Optogenetic advances as well as demonstration of their safety and efficacy in human gene therapy will likely parallel the technological improvements in the oCI.

This work represents a notable step forward on a long road to moving oCI closer to clinical use in humans by demonstrating small‐scale μLED‐based light sources that successfully evoke activity in auditory neurons with the ability to encode sound intensity and improved spectral selectivity over eCIs.
